# A Bibliometric Analysis of Publications on Pluripotent Stem Cell Research

**DOI:** 10.22074/cellj.2015.512

**Published:** 2015-04-08

**Authors:** Changshuan L. Lin, Yuh-Shan Ho

**Affiliations:** 1 Changshuan L. Lin Consulting, LLC, PHC, 1010 Race Street, Philadelphia, PA 19107 USA; 2 Trend Research Centre, Asia University, 500 Lioufeng Road, Wufeng, Taichung County 41354, Taiwan

**Keywords:** Pluripotent Stem Cells, Embryonic Stem Cells, Stem Cells

## Abstract

**Objective:**

Human pluripotent stem cells are self-renewing cells with the ability to differentiate into a variety of cells and are viewed to have great potential in the field of regenerative medicine. Research in pluripotent stem cells holds great promise for patient specific therapy in various diseases. In this study, pluripotent stem cell articles published from 1991 to 2012 were screened and retrieved from Science Citation Index Expanded (SCI-EXPANDED).

**Materials and Methods:**

In this retrospective study, the publication trend, citation trends for top articles, distributions of journals and Web of Science categories were analyzed. Five bibliometric indicators including total articles, independent articles, collaborative articles, first author articles, and corresponding author articles were applied to compare publications between countries and institutions.

**Results:**

The impact of top articles changed from year to year. Top cited articles in previous publication years were not the same as recent years. "Induced pluripotent stem cell (s)" and "embryonic stem cell (s)" were the most used author keywords in pluripotent stem cell research. In addition, the winner of the Nobel Prize in physiology or medicine in 2012, Prof. Shinya Yamanaka, published four of the top ten most frequently cited articles.

**Conclusion:**

The comprehensive analysis of highly cited articles in the stem cell field could identify milestones and important contributors, giving a historic perspective on scientific progress.

## Introduction

The historical origins of pluripotent stem cell research can be dated back to unpublished findings on the activity of stem cells and also the study of teratocarcinomas in 1950s (1). Cells with the capacity to form into any cells needed by the body are known as pluripotent cells (2). Pluripotent cells have the developmental potential to form derivatives of all three embryonic germ layers, including gut epithelium (endoderm); cartilage, bone, smooth muscle, and striated muscle (mesoderm) and neural epithelium, embryonic ganglia, and stratified squamous epithelium (ectoderm) (3). 

Later in the 1960s it was observed that a number of cells in a bone marrow suspension which were capable of continued proliferation (4,5) behaved as stem cells. The family of pluripotent stem cell lines has grown to include embryonal carcinoma cells, embryonic stem cells, embryonic germ cells, and now induced pluripotent stem cells (6). 

In the 1960s, pluripotent stem cell function of mouse marrow "lymphocyte" (7) was reported by three authors from the Oak Ridge National Laboratory in the United States (USA) along with the effect of hemopoietic organ stroma on differentiation of pluripotent stem cells (8). Pluripotent stem cells became a research focus in the following years. Subsequently, more research concerning pluripotent stem cells included characterization of pluripotent stem cell line derived from mouse embryo (9); hemopoietic pluripotent stem cells, defined as spleen colony-forming units (CFU-S), considered responsible for the generation of the hematopoietic system (10) and evidence for pluripotent stem cell origin of idiopathic myelofibrosis (11). In the early 1990s, Chaudhary and Roninson (12) found that the highest levels of permeability glycoprotein among the progenitors were associated with cells displaying characteristics of pluripotent stem cells.

Bibliometrics is a widely used tool to map the literature around a research field. The most recent bibliometric analysis of stem cell research was reported in 2003 (13). However, information from a variety of words in article titles, author keywords, and *KeyWords Plus* used to evaluate research trends in global stem cell research was published in 2009 (14). It was concluded that the application of stem cell transplantation technology to human disease therapy had become the orientation of all stem cell research in the 21^st^ century. In 2012, several bibliometric analyses of applications relating to stem cell transplantation technology were reported for diseases such as Parkinson’s disease (15), cerebral ischemia (16), spinal cord injury (17), Duchenne muscular dystrophy (18) and epilepsy (19). A sharp increase in stem cell research has been observed since 1991 (14). Not only have publicly and privately funded scientists been working in the field of stem cells, multi-national government funded policies have become equally as influential, for instance, the impact of federal funding policy (20), the Japanese government (14, 21) and Canadian research policy (22).

In this research, the pluripotent stem cell literature published from 1991 to 2012 was screened, and highly cited articles in total citations as well as citations in last year and publication year were identified and compared for impact in literature.

## Materials and Methods

Data used in this retrospective study were retrieved from the Thomson Reuters Web of Science, the online version of the Science Citation Index Expanded (SCI-EXPANDED) on 11 December 2013. The database was searched under the keywords "pluripotent stem cell" and "pluripotent stem cells" in terms of topic (title, abstract, author keywords, and *KeyWords Plus*) within the publication year with a limit of 1991 to 2012. *KeyWords Plus* supplied additional search terms extracted from the titles of articles cited by authors in their bibliographies and footnotes in the Institute for Scientific Information (ISI; now Thomson Reuters, New York) database, and substantially augmented title-word and author-keyword indexing (23). Non-article-type documents such as reviews, meeting abstracts, editorial materials, proceedings papers, letters, book chapters, news items, corrections, and notes were excluded. The final filter was the front page, in which only the articles having the search keywords in their first page including article title, abstract, and author keywords were retained (24). The impact factor of a journal was based on the Journal Citation Report 2012. The number of citations of an article in a single year, for example 2012, was referred to as the C2012 and the total number of citations since publication to 2012 was referred to as the TC2012 (25, 26). The collaboration type was determined by the addresses of the authors. Collaboration could be classified as either a single-country article, in which all authors’ addresses were from the same country, or an international collaborative article, which was co-authored by researchers from multiple countries (27). The records were downloaded and reorganized using Microsoft Excel 2010. In the SCI-EXPANDED database, the corresponding author was designated as the "reprint" author; this study instead used the term "corresponding author". In a single author article where authorship was unspecified, the single author was both first author and corresponding author. Similarly, for a single institution article, the institution was classified as the first author’s institution and the corresponding author’s institution. Affiliations in England, Scotland, Northern Ireland, and Wales were reclassified as being from the United Kingdom (UK) (27). Affiliations in Czechoslovakia were checked to be in Czech Republic and Slovakia. Affiliations in Hong Kong before 1997 were included with China (24).

## Results

There were 2,844 articles that met the selection criteria mentioned. Ninety-eight percent of the articles were published in English. Other languages also appeared, such as French (15 articles; 0.53% of 2844 articles), German (10 articles; 0.35%), Polish (6 articles; 0.21%), Korean (6 articles; 0.21%), Chinese (3 articles; 0.11%) and one for each in Czech, Japanese, Persian, Slovenian and Spanish, respectively.

The 2,844 articles were authored by 12,914 authors, among whom 9,648 (75%) contributed to only one article, 1,790 (14%) contributed to two articles, 653 (5.1%) contributed to three articles, 326 (2.5%) contributed to four articles, 159 (1.2%) contributed to five articles and 338 (2.6%) contributed to six or more articles. Moreover, 126 articles (4.4%) were single-author articles, 250 (8.8%) had two authors, 257 (9.0%) had three authors, 254 (8.9%) had four authors, 293 (10%) had five authors, 257 (9.0%) had six authors, 247 (8.7%) had seven authors, 233 (8.2%) had eight authors, 211 (7.4%) had nine authors, 154 (5.4%) had ten authors and 562 (20%) had more than ten authors.

### Trends in pluripotent stem cell publication

**Fig.1:**
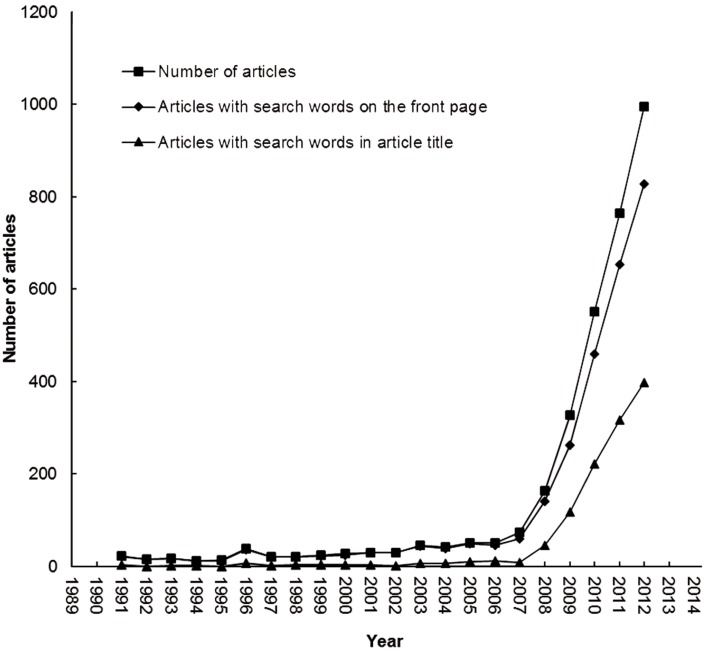
Publication trends from 1991-2013.

Figure 1 shows publication trends with articles searched by keywords "pluripotent stem cell" and "pluripotent stem cells" in terms of topic (title, abstract, author keywords, and *KeyWords Plus*), front page (title, abstract, author keywords), and article title only. In recent years, more pluripotent stem cell related articles have been published and the keywords are not always found in the article titles. Number of articles located via SCI-EXPANDED and the number of articles with the required keywords somewhere in the front page of the article were not the same. The rapid increase in the number of articles published after 2007 can be attributed to a couple of groundbreaking articles on pluripotent stem cell research. In 2006 and 2007, Takahashi at Kyoto University of Japan published as first author in Cell, articles entitled "Induction of pluripotent stem cells from mouse embryonic and adult fibroblast cultures by defined factors" (28) and "Induction of pluripotent stem cells from adult human fibroblasts by defined factors" (29). Within the same time period followed literature such as, "Induced pluripotent stem cell lines derived from human somatic cells" (30), "Generation of germline-competent induced pluripotent stem cells" (31) and "In vitro reprogramming of fibroblasts into a pluripotent ES-cell-like state" (32).

### Citation life cycles of articles

**Fig.2:**
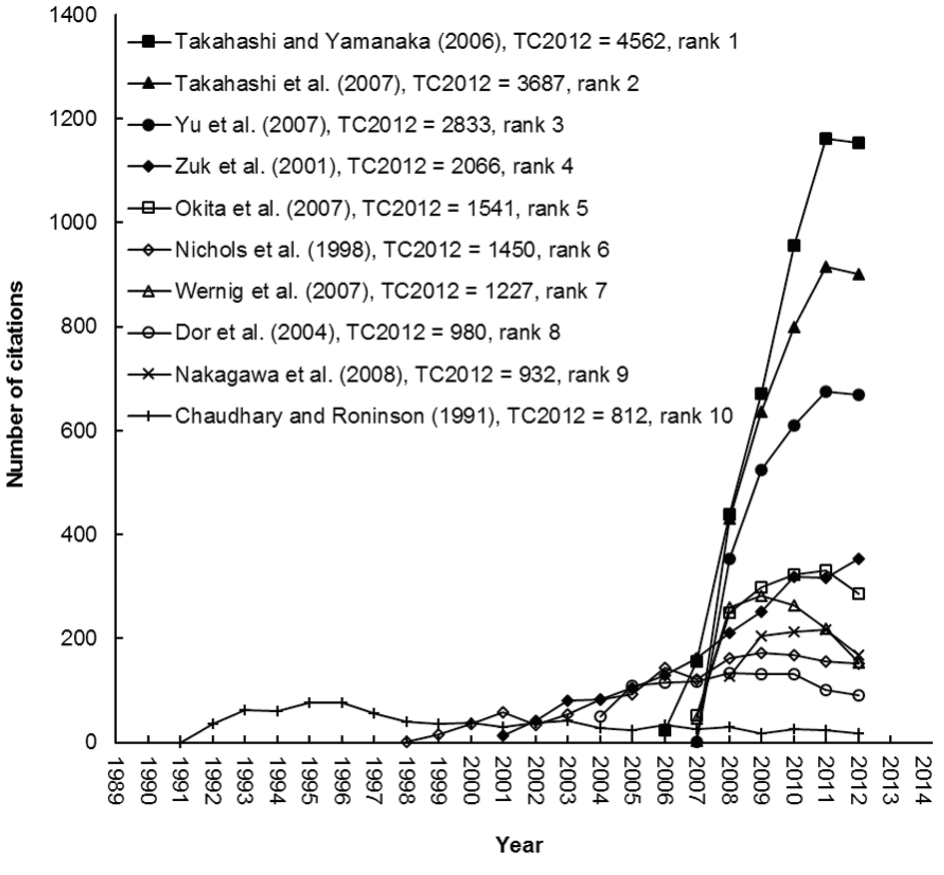
The lives of the top ten most frequently cited articles.

**Fig.3:**
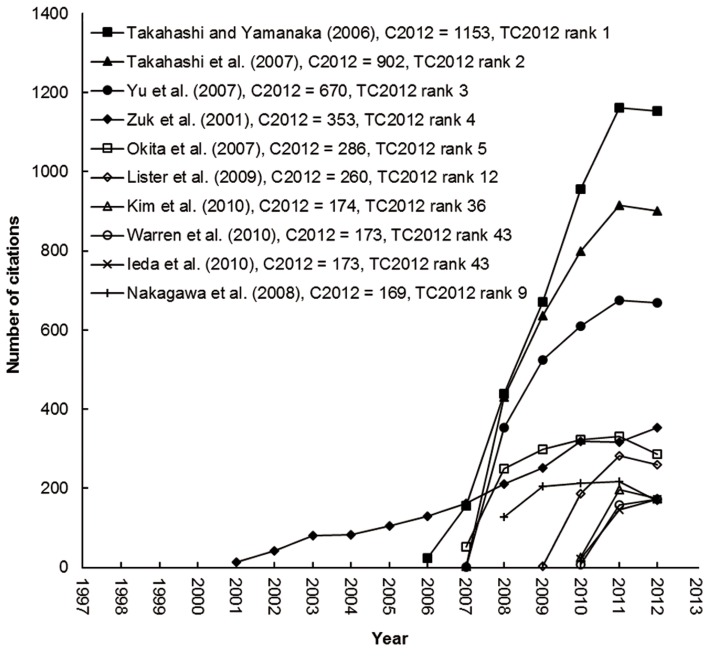
The lives of the top ten most frequently cited articles in 2012.

Table 1 presents the top articles cited in TC2012. Out of these 10 articles, seven were published after 2003 and one in 1991, 1998 and 2001, respectively. The journals in which these articles were published were Cell [impact factor (IF2012=31.957)] with four articles, and Nature (IF2012=38.597) with three articles, and one each in Nature Biotechnology (IF2012=32.438), Science (IF2012=31.027) and Tissue Engineering (IF2012=4.065). The citation index of an article might not be a direct measure of its quality or importance; it is a measure of recognition that may suggest its visibility or impact on the scientific community (33). Some scientists previously studied the citation life cycles of highly cited articles (34, 35). The articles with top TC2012 were recently reported (24). The citation lives of the top ten articles are shown in figure 2. The articles with the highest TC2012 can be considered the most popular articles in the last 20 years. In general, only the trends of the top three articles by Takahashi and Yamanaka (28) in 2006, Takahashi et al. (29) in 2007, and Yu et al. (30) in 2007 saw continuous sharp increases since their publication years, while the slopes of the other publications climbed initially but then reached a plateau after which they continued to be of high impact with more than 100 annual citations. However, the article by Chaudhary and Roninson (12) in 1991, which reached its peak citation rate five years alter its publication, subsequently decreased to 17 citations in 2012. Highly cited articles may not always be those with the highest impact (36). Some of the top ten highly cited articles in 2012 had just been published, for example in 2009 (25). Their citation lives are shown in figure 3. The articles with the highest C2012 were considered those with the most impact in the past few years. Recently published articles, for example, "Hotspots of aberrant epigenomic reprogramming in human induced pluripotent stem cells" (37) and "Somatic coding mutations in human induced pluripotent stem cells" (38) have great potential, but they did not have a high TC2012 because the time span was not sufficient to accumulate a large number of citations. Due to this, it has been suggested that the citations to an article within each individual year should be used instead (24). Almost all articles in figure 3 had high annual citation growth rates and thus great vitality, as evidenced by Takahashi and Yamanaka (28), Takahashi et al. (29), Yu et al. (30) and Zuk et al. (39) which have very high citation counts; TC2012>2,000 and C2012>350. However, three recently published articles by Kim et al. (40), Warren et al. (41) and Ieda et al. (42) influenced a great number of scientists with which resulted in a C2012>170, but a TC2012<400. Although they did not have enough time to accumulate high total citation counts, all of them have had a steep increase in citations since their papers were published.

**Table 1:** Ten most frequently cited pluripotent stem cell research articles in Science Citation Index Expanded


Rank (TC2012)	Rank (C2012)	Rank (C0)	Article

**1(4.562)**	1(1,153)	40 (24)	Induction of pluripotent stem cells from mouse embryonic and adultfibroblast cultures by defined factors (28).
**2(3.687)**	2(902)	319 (4)	Induction of pluripotent stem cells from adult human fibroblasts bydefined factors (29).
**3(2.833)**	3(670)	797 (1)	Induced pluripotent stem cell lines derived from human somatic cells (30).
**4(2.066)**	4(353)	82 (13)	Multilineage cells from human adipose tissue, implications for cell-basedtherapies (39).
**5(1.541)**	5(286)	13 (53)	Generation of germline-competent induced pluripotent stem cells (31).
**6(1.450)**	15(152)	552 (2)	Formation of pluripotent stem cells in the mammalian embryo depends onthe POU transcription factor Oct4 (64).
**7(1.227)**	14(154)	17 (47)	In vitro reprogramming of fibroblasts into a pluripotent ES-cell-like state (32).
**8(980)**	39(91)	14 (50)	Adult pancreatic beta-cells are formed by self-duplication rather thanstem-cell differentiation (65).
**9(932)**	10(169)	1 (128)	Generation of induced pluripotent stem cells without MYC from mouseand human fibroblasts (44).
**10 (812)**	329 (17)	1292 (0)	Expression and activity of P-glycoprotein, a multidrug efflux pump, inhuman hematopoietic stem-cells (12).


TC2012; Number of citations till 2012, C2012; Number of citations in 2012 and C0; Number of citations in publication year.

It is evident that articles published earlier are at an advantage in having time to gain more citations, compared to those published more recently (43). A total of 1,553 articles (55% of 2,844 articles) had no citations in the year of publication (C0=0) and 28 articles (0.99%) had more than 30 citations (C0>30) including one article in 2012, nine articles in 2011, and four articles in 2010. Articles with higher numbers of citations in the year of publication (C0) were likely to continue rising in later years. One of the reasons for this might be that the number of journals in the SCI-EXPANDED database increased from 4,963 in 1997 to 8,471 in 2012. The article published by Nakagawa et al. (44) itself had 128 citations in its year of publication. Increasing trends in citations after the year of publication or in later years could not be found for the majority of the top ten articles. The article entitled "Generation of induced pluripotent stem cells without MYC from mouse and human fibroblasts" by Nakagawa et al. (44) was the only article ranked in the top ten for the categories of TC2012, C2012 and C0. It is not uncommon that articles with high a TC2012 or C2012 have a low C0 (45).

### Journals and web of science categories

In total, 2,844 articles were published in 689 journals and were listed in 95 Web of Science categories in the science edition in 2012. According to Bradford’s Law of Scattering (46), the journals were sorted in descending order in terms of number of articles, and then divided into three "zones". Zone one represents the most productive one-third of the total articles, with 11 journals (1.6% of 689 journals). Zone two represents the next most productive one-third of total articles, with 85 (12%) journals and Zone three represents the least productive one-third of total articles with 593 (86%) journals. The number of journals was approximately 1: n: n2 (1: 7.7: 54), following Bradford’s law. The 11 most productive of Bradford’s core journals were Stem Cells (198 articles; IF2012=7.701), PLoS One (197 articles; IF2012=3.730), Stem Cells and Development (97 articles; IF2012=4.670), Proceedings of the National Academy of Sciences of the United States of America (93 articles; IF2012=9.737), Cell Stem Cell (84 articles; IF2012=25.315), Stem Cell Reviews and Reports (58 articles; IF2012=4.523), Nature (56 articles; IF2012=38.597), Blood (44 articles; IF2012=9.060), Journal of Biological Chemistry (41 articles; IF2012=4.651), Nature Protocols (37 articles; IF2012=7.960), and Biochemical and Biophysical Research Communications (36 articles; IF2012=2.406). The IF of a journal is defined by the Journal Citation Report (JCR). It is derived by dividing the number of current citations to articles published in the previous two years by the total number of papers published in the previous two years. It is a measure of the frequency with which the average paper in a journal has been cited in a particular year. The IF is used to evaluate a journal’s relative importance, especially when compared to others in the same field. Pluripotent stem cell research has been a topic which has expanded significantly since 2007 with more articles published in stem cell-related journals with higher IFs. It has also been reported that articles on other new research topics, such as severe acute respiratory syndrome-related research in the early stages (47).

The eight leading Web of Science categories which published at least 10% of all the articles were: cell biology with 835 articles (29% of 2,844 articles), followed by cell and tissue engineering with 594 (21%) articles, hematology with 475 (17%) articles, biotechnology and applied microbiology with 414 (15%) articles, multidisciplinary sciences with 393 (14%) articles, biochemistry and molecular biology with 252 (12%) articles, research and experimental medicine with 344 (12%) articles and oncology 294 (10%) articles.

### Publication by country

Research performance by country is measured using total number of articles, both independently written articles and collaborative articles (48). In recent years, five indicators have been used to measure research performance by country. These include total, independent, collaborative, first author, and corresponding author articles (49). The contribution provided by different countries was estimated from the affiliation of at least one author connected to the articles. There were nine articles without any address information on the Web of Science. Of the 2,835 articles with addresses, 2,105 (74%) articles were single-country articles and 730 (26%) articles were international collaborative articles. The top 20 countries were ranked by number of articles, including the number and percent of single country articles, international collaborative articles, first author articles, and corresponding author articles (Table 2). Two North American countries, nine European countries, eight Asian countries, and Australia were ranked in the top 20. The seven major industrialized countries of the world (G7) along with China, Spain, and South Korea were in the top ten. The G7 countries were highly productivity in terms of articles; including 2,248 (79% of 2,844) articles with affiliations. That advantaged countries dominate in terms of publications is not unsurprising as this pattern has already been seen for many medical-related topics, such as patent ductus arteriosus (50), asthma in children (51), stem cells (14), *Helicobacter pylori* (52) and human papillomavirus (53).

### Publication by institution

In recent years, indicators of publication performance of first authors (54), both first and corresponding author (36), institutions (55) and countries (56) have compared. To obtain more details, these indicators were used to compare publications by institution. The contributions of different institutions were defined by the affiliation of at least one author. Of the 2,835 articles with address information in the Web of Science, 1,012 (36%) were single institution articles and 1,823 (64%) articles were inter-institutional collaborations. Table 3 shows that among the top 21 institutions, 13 (62%) were in USA and four (19%) were in Japan. The leading institution was Harvard University in the USA, which published 147 pluripotent stem cell related articles from 1991 to 2012 in SCI-EXPANDED. Harvard University was also the institute with the most collaborations. However Harvard University ranked 13th on single institution articles and 5^th^ on first and corresponding author articles respectively. Stanford University in the USA published the most single institution articles, while Kyoto University of Japan had the highest number of first and corresponding author articles.

**Table 2:** Top 20 countries with at least 28 articles


Country	TP	TPR (%)	SPR (%)	CPR (%)	FPR (%)	RPR (%)

**USA**	1,329	1(47)	1(40)	1(66)	1(39)	1(39)
**Japan**	400	2(14)	2(14)	5(13)	2(13)	2(13)
**China**	306	3(11)	3(7.6)	3(20)	3(7.7)	3(7.8)
**Germany**	280	4(10)	4(6.2)	2(20)	4(6.7)	4(6.7)
**UK**	249	5(8.8)	5(5.8)	4(17)	5(6.6)	5(6.6)
**France**	127	6(4.5)	6(3.2)	7(8.1)	6(3.2)	6(3.1)
**Spain**	117	7(4.1)	13(1.4)	6(12)	8(2.4)	8(2.4)
**Canada**	116	8(4.1)	7(2.9)	8(7.4)	7(3.0)	7(3.0)
**South Korea**	98	9(3.5)	8(2.6)	10(6)	9(2.4)	8(2.4)
**Italy**	84	10 (3.0)	11 (1.6)	9(7.0)	10(1.7)	10(1.7)
**Australia**	72	11 (2.5)	9(1.8)	12(4.7)	11 (1.7)	11 (1.7)
**Israel**	59	12 (2.1)	10(1.7)	15(3.2)	12(1.5)	12(1.5)
**Netherlands**	59	12 (2.1)	16(0.81)	11 (5.8)	15(0.88)	16(0.82)
**Singapore**	54	14 (1.9)	14(1.1)	14(4.1)	14(1.2)	14(1.2)
**Sweden**	43	15 (1.5)	20(0.43)	12(4.7)	17(0.74)	17(0.75)
**Taiwan**	39	16 (1.4)	12(1.5)	24(1.1)	13(1.2)	13(1.2)
**Belgium**	29	17 (1.0)	20(0.43)	16(2.7)	17(0.74)	17(0.75)
**India**	28	18 (1.0)	16(0.81)	19(1.5)	17(0.74)	17(0.75)
**Iran**	28	18 (1.0)	15(1.1)	30(0.68)	15(0.88)	15(0.89)
**Poland**	28	18 (1.0)	19(0.48)	18(2.5)	22(0.49)	22(0.50)


TP; Total number of articles, TPR; The rank of number of total articles, SPR; The rank of single-country articles, CPR; The rank of number of internationally collaborative articles, FPR; The rank of number of first author articles, RPR; The rank of number of corresponding author articles and %; The percentage of each type articles among their total articles.

**Table 3:** Top 21 institutions with at least 36 articles


Institution	TP	TPR (%)	SPR (%)	CPR (%)	FPR (%)	RPR (%)

**Harvard University, USA**	147	1 (5.2)	13(0.89)	1 (7.6)	5 (1.6)	5 (1.6)
**Kyoto University, Japan**	132	2 (4.7)	2 (2.3)	2 (6.0)	1 (2.9)	1 (2.9)
**Chinese Academy of Sciences, China**	83	3 (2.9)	8 (1.1)	3 (3.9)	2 (1.8)	2 (1.8)
**University of California, San Diego, USA**	74	4 (2.6)	7 (1.4)	4 (3.3)	8 (1.2)	8 (1.1)
**Johns Hopkins University, USA**	69	5 (2.4)	3 (2.1)	7 (2.6)	3 (1.7)	3 (1.7)
**Stanford University, USA**	67	6 (2.4)	1 (2.7)	10 (2.2)	4 (1.6)	4 (1.6)
**Salk Institute for Biological Studies, USA**	58	7 (2.0)	44(0.4)	5 (3.0)	12 (0.81)	11 (0.86)
**Japan Science and Technology Agency, Japan**	55	8 (1.9)	169 (0.1)	5 (3.0)	166 (0.11)	161 (0.11)
**University of California, Los Angeles, USA**	53	9 (1.9)	4 (1.8)	15 (1.9)	6 (1.3)	6 (1.2)
**University of Wisconsin, USA**	49	10 (1.7)	8 (1.1)	11 (2.1)	8 (1.2)	8 (1.1)
**University of Cambridge, UK**	49	10 (1.7)	4 (1.8)	21 (1.7)	7 (1.2)	7 (1.2)
**Massachusetts Institute of Technology (MIT), USA**	47	12 (1.7)	44(0.4)	8 (2.4)	27 (0.49)	27 (0.50)
**University of Tokyo, Japan**	42	13 (1.5)	17(0.69)	15 (1.9)	11 (0.85)	11 (0.86)
**Massachusetts General Hospital, USA**	41	14 (1.4)	N/A	9 (2.2)	21 (0.63)	19 (0.64)
**University of California, San Francisco, USA**	41	14 (1.4)	8 (1.1)	22 (1.6)	12 (0.81)	14 (0.78)
**University of Toronto, Canada**	41	14 (1.4)	29(0.49)	12 (2.0)	25 (0.53)	25 (0.53)
**Max Planck Institute for Molecular Biomedicine,Germany**	41	14 (1.4)	23(0.59)	15 (1.9)	15 (0.74)	16 (0.71)
**Scripps Research Institute, USA**	37	18 (1.3)	44(0.4)	18 (1.8)	12 (0.81)	13 (0.82)
**Children’s Hospital, USA**	36	19 (1.3)	N/A	12 (2.0)	42 (0.35)	44 (0.36)
**Harvard Stem Cell Institute, USA**	36	19 (1.3)	N/A	12 (2.0)	N/A	N/A
**Osaka University, Japan**	36	19 (1.3)	65(0.3)	18 (1.8)	16 (0.71)	15 (0.75)


TP; Total number of articles, TPR; The rank of number of total articles, SPR; The rank of single-country articles, CPR; The rank of number of internationally collaborative articles, FPR; The rank of number of first author articles, RPR; The rank of number of corresponding author articles, %; The percentage of each type articles among their total articles and N/A: Not applicable.

### Research trends and hotspots

In recent years, the distribution of words in article titles, author keywords, and *KeyWords Plus* have been applied to evaluate trends in research topics (14). Detection of certain words in the abstracts of papers has also been used as information to determine research trends (57). Furthermore, "word cluster analysis" has been successfully applied to find the research hotspots for specific topics (58). All the articles have titles, while not all articles was listed their keywords. The sample size of articles with titles was now more comprehensive, because one article might have certain words in its title but might not be consistent with author keywords and *KeyWords Plus*. However, the meaning of individual words in a title is not always inclusive, therefore, there was need to refer to the author keywords for better understanding (59). Among 2,844 articles, 1,567 articles (55%) had recorded information on author keywords, while 2,774 articles (98%) with *KeyWords Plus* information were analyzed.

**Fig.4:**
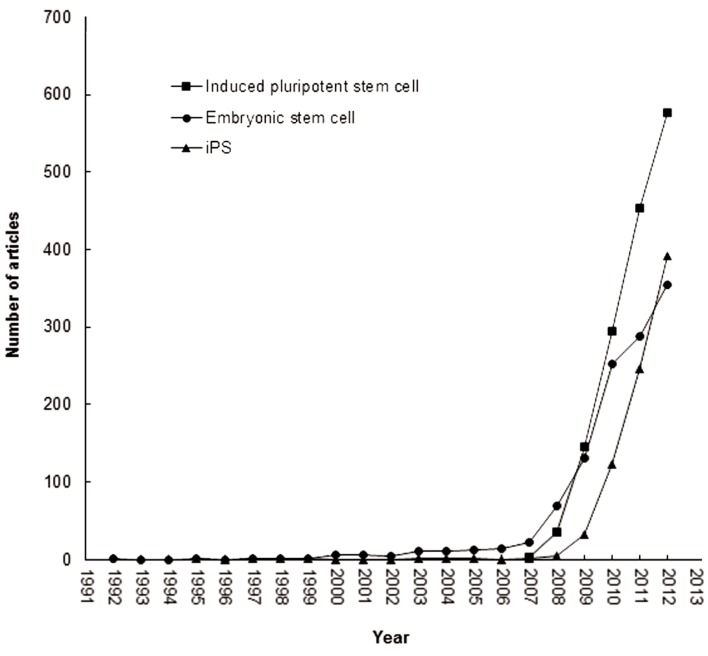
Growth trends of new keywords during 1991-2012.

The most frequently used author keyword was "induced pluripotent stem cells" and "induced pluripotent stem cell". In 2007, the 3rd ranked article based on keywords was titled "Induced pluripotent stem cell lines derived from human somatic cells" (30) which was written by 12 authors from Genome Center Wisconsin and University of Wisconsin in USA with TC2012=2,833 and C2012=670. Figure 4 shows a sharply increased trend in "induced pluripotent stem cell" research. "Embryonic stem cells" and "embryonic stem cell" ranked 2nd among the author keywords. "Embryonic stem cells" related articles were found as early as 1992, with continued increasing trends from 2007. "Differentiation", "reprogramming", "stem cells", "stem cell", and "pluripotency" were also keywords frequently used by authors. In addition, abbreviation of induced pluripotent stem cells such as "iPS cells" and "iPSC", along with induced pluripotent stem as "iPS", as well as "human induced pluripotent stem cells" are more newly used author keywords. Abbreviation of induced pluripotent stem cells, "iPS cells" first appeared in the abstract of the article titled "Induction of pluripotent stem cells from mouse embryonic and adult fibroblast cultures by defined factors" (28). The abbreviation has become acceptable for use in pluripotent stem cell research and is gaining popularity (Fig.4).

## Discussion

In 2000, the Japanese government released a report that had been on hold for a long period of time. The report endorsed the use of human stem cells in research-work (21). The draft report outlined a process for both publicly and privately funded scientists to follow in deriving and working with stem cells. However, the number of stem cell research collaborations in the UK and USA has not been affected by the different national stem cell policies or regulatory mechanisms that motivates international stem cell research including in other countries with which the USA and UK most often collaborate (60). Prof. Shinya Yamanaka of Kyoto University of Japan and the Gladstone Institutes, USA received the Nobel Prize in physiology or medicine in 2012 for the discovery that mature cells can be reprogrammed to become pluripotent. In addition, Yamanaka has published 57 pluripotent stem cell articles of which four articles ranked in the top ten for TC2012 and C2012, respectively, including the top ranked article entitled "Induction of pluripotent stem cells from mouse embryonic and adult fibroblast cultures by defined factors" (28) and 2^nd^ ranked article entitled “Induction of pluripotent stem cells from adult human fibroblasts by defined factors" (29). A steep slope could be found with these two distinguished patterns of citations per year. It has been noticed that in the highest percentile, the top 0.1% of authors, a significant percentage have won the Nobel Prize or eventually proceeded to win the award in later years (61). Nobel Prizes are the gold standard of quality in scientific achievement in the fields where they are given (62). Furthermore, a high correlation between the bibliometric indicators and the number of Nobel Prize achievements was found in chemistry, medicine/physiology, and physics (63).

## Conclusion

This bibliometric investigation of articles on pluripotent stem cell-related research has revealed some interesting findings. In total, 2,844 articles were published in SCI-EXPANDED from 1991 to 2012. Articles were published in 689 journals and were listed in 95 Web of Science categories in the science edition in 2012. Stem Cells and PLoS One were the most common journals in pluripotent stem cell research. Mainstream research was in Web of Science category cell biology. There was a sharp increase in articles annually after 2007. Articles without the required search words on their front page could be still found in SCI-EXPANDED which is designed for researchers to find literature but not for bibliometric study. The citation lives of the top articles in total citations as well as in publication year and recent year showed that the impact of top articles in a research field might alter according to novelty and not only time. In general, the so-called "classic" articles had low citations in their publication year. The G7 were part of the top ten countries in terms of publication. It was noted that the USA, as a country, contributed the most independent and internationally collaborative articles, as well as the most first and corresponding author articles. Institutionally, Harvard University published the most inter-institutional articles and overall articles, while Kyoto University published the most first and corresponding author articles. The papers of the Nobel Prize winner in 2012, Prof. Shinya Yamanaka, who published four articles ranked among the top ten in terms of total citations and citations in the most recent year, have followed a distinguished pattern with steep slopes indicating rapidly increasing citation counts. Studies on induced pluripotent stem cells and embryonic stem cells have been found to be the most popular research focus in recent years in pluripotent stem cell research. As seen by the popularity of stem cell research in various countries and the acknowledgment of the potential of pluripotent stem cells to aid in patient specific therapies in our ever increasing elderly population, regenerative medicine will continue to progress with the advent of new research findings.
